# Blood-Letting, a Valuable Curative Means, Etc.

**Published:** 1873-07

**Authors:** James Rodgers

**Affiliations:** Knoxville, East Tennessee


### BLOOD-LETTING, A VALUABLE CURATIVE MEANS, TOO MUCH DISREGARDED.

BY JAMES RODGERS, M.D., KNOXVILLE, EAST TENNESSEE.

[Read Before the Knox County Section of the East Tennessee Medical Society.]

Blood-letting, as a remedial agent in the treatment of many of the diseases to which the human family are subject, originated in the early ages of the world. It seems to have been practiced by savage nations, doubtless suggested by the immediate relief following the spontaneous or accidental discharge of blood, hence they seem to have learned a very important lesson in the treatment of some of the diseases to which they were subject.

It is a well known fact that a spontaneous flow of blood from the nose will often relieve inflammation of the brain, and a hemorrhage, by some accident, will occasionally remove a chronic inflammation. We should learn from this the valuable lesson that nature, in her effort to relieve, always dictates the proper course.

There are a few simple and powerful remedies that are employed for the removal of all inflammatory affections; the first and most valuable of them is, unquestionably, the use of blood-letting. It is one of the most powerful of all our remedies, and requires much care and caution in its use. If we neglect this remedy in cases where it is demanded, we allow the disease to make dangerous progress; on the other hand, if we resort to it inappropriately, we will do much harm. It, therefore, becomes our duty to exercise the greatest amount of care, so that our patients may have the full benefit of this important, I would say indispensable, remedy. I will briefly allude to some of the diseases to which I consider blood-letting particularly adapted.

Inflammations, particularly of the serous membranes and of the substance of organs, and designated as pleuritis, peritonitis, arachnitis, pneumonia, hepatitis, etc., are among the diseases that require prompt, and oftentimes repeated, blood-letting, in order to afford timely relief.

Inflammatory fevers are often cut short by the judicious use of the lancet; and in every form of fever the expediency of bleeding is to be considered. The remedy should not be indiscriminately employed, but the circumstances of each case must be weighed before we decide to employ or withhold the lancet. Should local congestion or inflammation of some of the vital organs supervene, blood-letting, both general and local, becomes the most potent remedy. But every patient who is seized with a fever should not be bled indiscriminately. Regard must be given to age, to constitutional powers, to previous habits, and to mode of living. Without discrimination, doubtless, many valuable lives would be sacrificed. But, on the other hand, if duly cautious in our investigations, we need not err in the use of this anchor of safety.

Puerperal fever is a fatal form of disease in which the use of the lancet is attended with marked benefit, especially if employed in the early inflammatory stage. The celebrated John Armstrong speaks in strong terms of the importance of the lancet in this disease, and its use is urged also by Churchill, Watson, Drake, and many others. I invariably use the lancet in puerperal states, if the pulse is quick and hard, and abdominal pain and tenderness present, and I am not aware that it has failed, in a single instance, to afford much comfort, if it did not entirely arrest the progress of the disease. In acute rheumatism, I have often resorted to blood-letting with good results, especially where febrile symptoms were of a high grade, with a quick hard pulse.

In neuralgia, local bleeding by cups or leeches often affords much relief.

Acute inflammation of the mucous membranes often run on to a fatal termination very rapidly, unless promptly relieved. Laryngitis, bronchitis, enteritis, and other forms of inflammatory affections often become very serious in character, unless they are promptly met by some of the known remedial agents, and I am free to assert that I know of none that promises more speedy relief than blood-letting, both general and local. I have seen good results from blood-letting in the early stage of small-pox, when the fever was high—preceding the eruptive stage. It lessens irritation, relaxes the skin, and does much toward preventing congestion and inflammation. Armstrong says: "In the first form, which is an open inflammatory form of eruptive fever, the affection is of a highly inflammatory character; the fever is fully developed, with a skin intensely hot and dry, and a quick, bounding pulse; face flushed; tongue considerably furred; face swells rapidly. Bronchial complications often supervene, and the powers of life give way gradually or suddenly." With such a state of facts it is very clear to my mind that blood-letting promises more speedy relief than any other remedy; in fact I would feel, if I withheld the lancet, that I was guilty of unpardonable neglect.

In cynanche trachealis, I have often seen the most prompt benefit from the use of the lancet, or cups, if employed in the early stage, where the patient is strong and plethoric. Under such circumstances, I think of no other remedy that promises more relief in so short a time.

I might continue to enumerate many other forms of disease in which the use of blood-letting has been found to act promptly in alleviating, and in many cases removing at once, the violent symptoms. Suffice it to say that, in my humble opinion, there is a period in the progress of all inflammatory fevers, and also all other diseases putting on a high degree of local irritation or inflammation, during which we can not err in the use of blood-letting, either general or local, or both, as the emergency of the case demands.

We accomplish two objects by the use of this remedy: first, we lessen the force of the heart's action; and second, we ease the engorged capillaries of the part inflamed, and prepare the system for the immediate use of auxiliary remedies, if such be required.

If a morbid change in the character of the blood, produced by the introduction of a poison, be the cause of inflammatory fevers—which theory we adopt, for the reason that we think it is clearly shown by pathologists in treating of the fluids—"that the blood is not only materially changed in fever, but that the diseased state precedes the attacks, and that the changes take place in a determinate order." This view is corroborated by Andral, who says. "that the fever termed inflammatory seems often to arise from no other source than the blood being too rich in fibrin. In like manner, an impoverished state of the blood, whether accidental or natural, is often connected with mucous fevers, and with those characterized by a sudden sinking of the vital powers, and that the source and primary seat of typhus fevers, properly so called, is proved to be in the blood, inasmuch as they are caused by the introduction of deleterious substances, such as animal or vegatable effluvia, into that fluid. It is in this way only, by the blood becoming contaminated, and in this state circulating through the system, that fever can be supposed to be, according to the language of Dr. Fordyce, "a general disease which affects the whole system, both body and mind."

In fevers which are produced by living in an impure atmosphere, the blood becomes the medium through which the morbific matter is circulated through the system, the deleterious effects of which have been clearly demonstrated by the experiments of Magendie, and to which I need only refer.

If this condition of the blood be the cause of inflammatory fevers, it becomes our first duty to remove the cause of the malady as speedily as possible, and this can not be done in any other way, as readily and with as much safety to the patient as by the use of the lancet.

In addition to the direct benefits derived from blood-letting, we must take into account its modifying influence on the action of other remedies. Emetics, cathartics, sudorifics, antiphlogistics, etc., can not be successfully employed while the arterial excitement is above a certain point; but they act kindly after the use of the lancet. We feel much inclined to adopt the humoral pathology as taught by Hippocrates, Galen, Celsus, and Sydenham, viz: That fever is produced by the presence of noxious substances in the blood; not only in the case of fevers, but in all other diseases, the blood becomes contaminated or depraved, either by the reception of noxious substances from without, or in consequence of changes going on within the body; that the system is excited by the presence of these impurities, either to direct efforts for their expulsion, or to a course of elaborative action, so as to fit them for expulsion; and that, in the language of Sydenham "a disease is no more than an effort of nature to throw off the morbific matter, and thus recover the patient." This is the sum and substance of the humoral pathology. According to this view, fever is only a violent effort of the system to rid itself of noxious matter, and the sweats, bilious discharges, turbid urine, suppurations, and cutaneous eruptions which so often attend fevers, are merely different means by which this matter is expelled. We do not feel prepared to adopt the humoral pathology as a whole, but in so far as it relates to inflammatory fevers, we think it correct. The doctrine taught by Hoffman, "That to the nervous system belongs the source of mischief," is doubtless correct in many cases. I do not propose to investigate the many different theories in reference to the pathology of diseased action, but to call attention to that remedy which at one period was so highly extolled in the treatment of all inflammatory diseases, but which has been so much neglected of late by a majority of medical men. A very intelligent practitioner said to me: "If a physician was to bleed a member of my family under any circumstances, I would sue him for malpractice." Such erroneous teachings are well calculated to do much harm, by prejudicing the community against the use of the lancet, and it is much to be deplored that so many of our intelligent medical men have discarded or neglected this very important remedial agent in the treatment of inflammatory fevers, and other diseases attended with irritation or inflammation.

